# Patient-Generated Subjective Global Assessment (PG-SGA) predicts length of hospital stay in lung adenocarcinoma patients

**DOI:** 10.1017/S0007114521003500

**Published:** 2022-05-28

**Authors:** Jilu Lang, Yanan Shao, Jiehao Liao, Jia Chen, Xuewen Zhou, Rong Deng, Wei-Jan Wang, Xian Sun

**Affiliations:** 1 Department of Cardiac Vascular Center, The Seventh Affiliated Hospital, Sun Yat-Sen University, Shenzhen, People’s Republic of China; 2 Department of Cardiovascular Surgery, The Second Affiliated Hospital of Harbin Medical University, Harbin, People’s Republic of China; 3 Department of Medical Oncology, Harbin Medical University Cancer Hospital, Harbin, People’s Republic of China; 4 Department of Oncology, The Seventh Affiliated Hospital, Sun Yat-Sen University, Shenzhen, People’s Republic of China; 5 Department of Biological Science and Technology, Research Center for Cancer Biology, China Medical University, Taichung, Taiwan

**Keywords:** Nutrition assessment, Length of hospital stay, Lung cancer

## Abstract

The prevalence of malnutrition is high among oncology patients in Northern China. Malnutrition is related to the longer hospital stay, and it can be used to predict the prognostic outcome of patients. This work focused on investigating the relationship of nutritional condition with the length of hospital stay (LOS) in Northern Chinese patients with lung adenocarcinoma (LUAD). The Patient-Generated Subjective Global Assessment (PG-SGA), Nutritional Risk Screening 2002 (NRS 2002) score, recent weight loss and BMI were assessed in a probabilistic sample of 389 LUAD patients without epidermal growth factor receptor (EGFR) mutations. This study collected the demographic and clinical features of patients in a prospective manner. Then, we examined the association of nutritional status with LOS among the population developing LUAD. According to the PG-SGA, 63 (16·3 %), 174 (44·7 %) and 78 (20·1 %) patients were at risk for undernutrition, moderate undernutrition and severe undernutrition, respectively. Nutritional risk was found in 141 (36·2 %) patients based on the NRS 2002. The average LOS for tumour patients in Northern China was 12·5 d. At admission, a risk of undernutrition or undernutrition according to the PG-SGA (*P* < 0·001), NRS 2002 (*P* < 0·001) and latest weight loss (*P* < 0·001) predicted the longer LOS. LOS was related to nutritional status and hospitalisation expenses (*P* < 0·001). LUAD patients who stayed in the ICU had a poorer nutritional status and a longer LOS (*P* < 0·001). In Northern Chinese patients with LUAD, a risk for undernutrition evaluated by the PG-SGA, the NRS 2002 and recent weight loss, but not BMI, could predict a longer LOS.

Non-small cell lung cancer accounts for a main reason leading to cancer-associated mortality in the world. Advanced lung cancer patients with no driver mutation are most commonly treated with platinum-based chemotherapy. Length of stay (LOS) can be adopted to be an alternative outcome indicator for hospitalisation-induced health changes or a marker to predict the well-being of a patient. The prediction of LOS contributes to developing the efficient hospital resources and health care plan. As reported previously, malnutrition assessed using the Nutritional Risk Screening 2002 (NRS 2002), Malnutrition Universal Screening Tool (MUST) and Subjective Global Assessment (SGA) predicts the prolonged LOS^(2–4)^. The relationships between nutritional status and LOS in lung adenocarcinoma (LUAD) patients are not known.

It has been suggested that LOS is affected by numerous variables, including age, disease type and severity, and nutritional status. As a result, the existing findings can be biased since such variables are not taken into comprehensive consideration^(5,6)^. So it is essential to study the relationships in Northern Chinese LUAD patients with adjustments for tumour node metastasis (TNM) classification and tumour grade as potential confounders. LOS predicts patient prognostic outcome and is usually adopted to be the outcome marker. Furthermore, predicting LOS has been indicated as the vital task for the medical team to develop a comprehensive health care plan or to efficiently manage the hospital resources. LOS is directly related to hospitalisation expenses. Several factors, including patient-associated variables, are determinants of LOS, like diagnosis, age or treatment in the hospital^(7,8)^.

Notably, both SGA and Patient-Generated Subjective Global Assessment (PG-SGA) have been the recognised approaches to assess nutritional condition for determining LOS in people suffering from gastrointestinal cancer^(9)^. As reported by NRS 2002, compared with cases with no nutritional risk, those with nutritional risk are associated with the prolonged LOS. Our results in this work indicated that both NRS 2002 and PG-SGA showed close relationships with the age-independent discharge time. Nutritional deficiency is more commonly found in cancer patients than in the general population, and it has been estimated that 16 % of Chinese cancer patients can be classified as malnourished^(10)^.

The practice guidelines recommend that PG-SGA can be an approach to assess nutritional condition in cancer patients^(11)^. This work focused on quantifying the relationships of BMI, PG-SGA-identified malnutrition and the NRS 2002-assessed malnutrition risk with LOS among different Northern Chinese inpatients with LUAD. In this study, we sought to analyse the data from Northern Chinese patients with LUAD for assessing the effectiveness and creditability of the present PG-SGA version for predicting LOS.

## Study design

All subjects in the present work were pathologically confirmed LUAD cases with no EGFR mutations. Patients at TNM stage IIIB and IV were deemed as advanced patients. This study adopted the eighth edition Lung Cancer TNM classification System (the 8th edition). Because all the patients were advanced LUAD without driver gene mutation, according to the National Comprehensive Cancer Network guideline, patients were treated with pemetrexed plus platinum chemotherapy. This study excluded patients receiving the optimal supportive care alone, or those suffering from impaired cognition or additional acute psychological disorders. The present work was approved by the Ethics Committee at Harbin Medical University Cancer Hospital and carried out strictly following the Declaration of Helsinki. Each participant provided the written informed consent for participation.

## Study sample

This study collected LUAD cases at stage IIIB and IV with the age of more than 18 years. All the enrolled patients provided the informed consent and stayed for over 24 h. Clock drawing test was used for cognitive impairment assessment. Patients are instructed to draw a round clock circle with two clock arms facing towards a given time point. The time used is between 2 and 10 min. All patients did not have the cognitive impairment. One trained investigator was responsible for collecting data. Data, such as age, sex, admission date and diagnosis, were obtained from the clinical documents. The body weight (BW) of each patient with light clothes was measured by the mechanical scale (precision, 0·1 kg), whereas the body height was determined using the fixed measuring tape (precision, 0·1 cm). The patients were weighed and measured by a nurse within the previous 48 h. BMI was calculated based on the measured BW and body height as follows, (BMI = BW (kg)/(body height (m))^2^). Thereafter, we adopted the NRS 2002 and PG-SGA for assessing the nutritional condition when patients were admitted. Each item of the PG-SGA was scored, while the eventual score stood for the necessity of nutritional intervention. Generally, PG-SGA can be rated subjectively. The physical examination, including body fluid status and fat and muscle stores, was conducted by a dietitian or physician. Each section was allotted a score, with higher scores representing a poorer nutritional status. To be specific, the patient is possibly ‘well nourished (0–1)’, at ‘nutritional risk (2–3)’, have ‘moderate undernutrition (4–8)’ or have ‘severe undernutrition (≥ 9)’. NRS 2002 categorises nutritional condition according to BMI, latest weight loss proportion, disease severity and latest alterations of food intake. Patients aged over 70 years had one more point. A patient was recognised to be associated with a nutritional risk if the eventual score reached ≥ 3 points. Patients with insufficient information (PG-SGA score or BMI) were excluded.

## Data analysis

Sample size was based on the correlation between screening tools and LOS of lung cancer. Based on the current investigation, the computing formula of sample size is as follows:

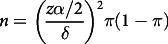




In the above formula, *n* is the sample size; Z*α*/2 is the *Z* value of two-sided test under the specified acceptable quality level–*α*. In this study, *α* = 0·05 and Z*α*/2 = 1·96. According to the documents, the probability of suffering from cancer diseases due to malnutrition is about 16 %, π = 16 %. The tolerance error *δ* = 5 %. According to the formula, *n* 207 can be obtained. The normally distributed variables were identified by Kolmogorov–Smirnov test. The data are presented as means and standard deviations. Cumulative probability of discharge with time was estimated based on BMI, NRS 2002 and PG-SGA score by Kaplan–Meier method. In this study, we censored patients whose LOS > 30 d (0·7 %, *n* 3) as 30 d. SPSS18.0 was adopted for statistical analysis. *P* < 0·05 indicated the significance level.

## Results

This work enrolled altogether 389 Northern Chinese cases suffering from LUAD. Table 1 presents patient features. The age of all enrolled cases was between 39 and 78 years. The LOS of women (12·1 (sd 7·4) d) was shorter than that of men (12·6 (sd 6·9) d). The nutritional state of women (PG-SGA: 5·5) was slightly better than that of men (PG-SGA: 6·3). Seventy-four percentage (*n* 288) of the patients were ≥ 65. Compared with patients < 65, patients ≥ 65 had a longer LOS and poorer nutritional state (12·4 (sd 5·5) *v.* 13·2 (sd 7·0); PG-SGA: 5·3 *v.* 7·6) (Table 1). Based on the size of the primary tumour, lymph nodes and distant metastasis, 32·9 % (*n* 128) of the tumours were classified as TNM stage III, and 67·1 % (*n* 261) were stage IV. Different metastatic sites were associated with different LOS and nutritional states. Patients with liver and brain metastasis and malignant effusions had longer LOS and poorer nutritional states. Tumour grade was not significantly related to PG-SGA (*P* = 0·387) (Table 1).

**Table 1.** Characteristics of the sample (Numbers and percentages; mean values and standard deviations)

			Length of stay (days)		PG-SGA
*n* 389	*n*	%	Mean	sd	
Weight (kg)					
Mean	62·43				
sd	12·4				
Length of stay (days)					
Median	12·5				
IQR	7·4–31·9				
Sex					
Women	192	49·4	12·1	7·4	5·5
Men	197	50·6	12·6	6·9	6·3
Age (years)					
Mean	58·13				
sd	11·8				
<65	288	74	12·4	5·5	5·3
≥ 65	101	26	13·2	7·0	7·6
Health insurance					
No	27	6·9	12·3	7·1	6·3
Yes	362	93·1	12·5	7·5	6·5
TNM classification					
III	128	32·9	10·7	6·7	5·8
IV	261	67·1	13·8	6·9	8·1
Metastatic sites					
Lung	37	9·6	12·1	6·5	5·7
Liver	81	20·7	14·7	7·2	7·3
Adrenal	30	7·7	12·2	7·1	4·9
Brain	53	13·7	14·6	7·9	7·7
Bone	88	22·6	12·8	6·1	4·8
Malignant effusions	98	25·2	14·9	5·7	8·7

IQR, interquartile range; NRS 2002, Nutritional Risk Screening; PG-SGA, Patient-Generated Subjective Global Assessment.

LOS prolonged remarkably with the nutrition condition and risk, including PG-SGA score, NRS 2002 score and BW changes (Table 2). As estimated by PG-SGA, there were 80·1 % cases with nutritional risk, including 20·1 % with severe malnutrition. As evaluated by NRS 2002, there were 36·2 % cases with nutritional risk (Table 2). There was a statistically significant association between PG-SGA score and LOS (*P* < 0·001). Concerning the NRS 2002, 3·2 % (*n* 12) of patients classified without nutritional risk had severe undernutrition (PG-SGA ≥ 9). A total of 19·8 % (*n* 77) of all the patients with a NRS 2002 score ≥ 3 also had a PG-SGA score ≥ 9. NRS was significantly related to LOS (*P* < 0·001). The number of patients with weight loss in the past 6 months (*n* 306) was greater than that (*n* 251) in the past 1 month. The number of patients with a stable weight in the past 6 months (*n* 62) was less than that (*n* 133) in the past 1 month. BW changes were significantly related to LOS (*P* < 0·001). A total of 14·0 % (*n* 55) of patients had a BMI ≤ 18·5, but BMI was not significantly related to LOS (*P* = 0·465).

**Table 2.** Median length of stay (LOS) according to nutritional risk and status at admission (Numbers and percentages; mean values and standard deviations)

	*n*	%	LOS (days)		*P*
			Mean	sd	
PG-SGA					
Well nourished or anabolic (0–1)	74	18·9	8·4	5·2	<0·001
Nutritional risk (2–3)	63	16·3	12·2	7·0	
Moderate undernutrition (4–8)	174	44·7	12·9	6·8	
Severe undernutrition (≥ 9)	78	20·1	13·9	7·1	
NRS 2002					
Without nutritional risk	248	63·8	11·9	7·0	<0·001
With nutritional risk	141	36·2	13·3	7·3	
Body weight changes					
Recent 1 month					
Weight loss	251	64·4	13·9	7·2	<0·001
Stable	113	29·1	12·6	7·1	
Weight gain	25	6·5	11·1	6·7	
Recent 6 month					
Weight loss	306	78·7	13·5	7·3	<0·001
Stable	62	15·9	11·8	7·4	
Weight gain	21	5·4	10·2	7·0	
BMI (kg/m^2^)					0·465
Underweight ≤ 18·5	55	14·0	12·7	7·3	
Normal weight 18·5–24·9	250	64·3	12·3	7·4	
Overweight/obesity ≥ 25	84	21·7	11·9	7·1	

NRS 2002, Nutritional Risk Screening; PG-SGA, Patient-Generated Subjective Global Assessment.

Among these patients, the TNM classification was significantly related to the PG-SGA score (*P* < 0·001). Patients classified as TNM stage III had a shorter LOS and better nutritional state (11·2 (sd 6·9) *v.* 13·7 (sd 7·7); PG-SGA: 5·7 *v.* 7·3). Only 3·2 % of the tumours (*n* 12) were classified as well differentiated (G1), while 36·2 % (*n* 141) were G2, 33·2 % (*n* 129) were G3 and 27·4 % (*n* 107) were G4; tumour grade was not statistically significantly to the PG-SGA score (Table 3).

**Table 3.** Patient-Generated Subjective Global Assessment (PG-SGA) and length of stay (LOS) according to TNM classification and tumour grade (Numbers and percentages; mean values and standard deviations)

	*n*	%	PG-SGA	LOS (days)		*P*
				Mean	sd	
TNM classification						
III	128	32·9	5·8	10·7	6·7	<0·001
IV	261	67·1	8·1	13·8	6·9	
Tumour grade						
G1	12	3·2	5·6	12·5	7·3	0·498
G2	141	36·2	6·0	11·9	5·4	
G3	129	33·2	6·9	13·0	6·9	
G4	107	27·4	6·2	12·3	6·5	

LOS was associated with hospitalisation expenses and alterations of nutritional condition. For patients with LOS < 30 d, the better the nutritional status, the lower the hospitalisation expenses. The average hospitalisation expenses were 23 768·3 (Table 4). A total of 49·8 % (*n* 194) of the hospitalisation expenses were ≤ 19 999. The PG-SGA score was statistically related to hospitalisation expenses. The hospitalisation expenses were ≤ 19 999, 20 000–39 999, 40 000–59 999 and ≥ 60 000 for average LOS of 9·2 (sd 4·8), 16·3 (sd 6·3), 16·7 (sd 6·6) and 20·3 (sd 6·2) d, respectively. LOS was significantly associated with hospitalisation expenses (Table 4).

**Table 4.** Patient-Generated Subjective Global Assessment (PG-SGA) and length of stay (LOS) according to hospitalisation expenses (Numbers and percentages; mean values and standard deviations)

Hospitalisation expenses (Renminbi, RMB)	*n*	%	PG-SGA	LOS (days)		*P*
				Mean	sd	
≤ 19 999	194	49·8	5·7	9·2	4·8	<0·001
20 000–39 999	78	20·1	5·8	16·3	6·3	
40 000–59 999	69	17·8	6·7	16·7	6·6	
≥ 60 000	48	12·3	7·1	20·3	6·2	

According to the physical examinations, LOS was associated with fluid status, muscle status and fat reserves. For patients who stayed fewer than 30 d in the hospital, LOS increased significantly with the PG-SGA score. The PG-SGA score was statistically related to the individual factors of fat stores, muscle status and fluid status (*P* < 0·001). A total of 11·2 % (*n* 44) of patients only had oedema, 1·0 % (*n* 4) only had ascites and 3·6 % (*n* 14) had both oedema and ascites (Table 5).

**Table 5.** Patient-Generated Subjective Global Assessment (PG-SGA) and length of stay (LOS) according to physical examination (Numbers and percentages; mean values and standard deviations)

Physical examination	*n*	%	PG-SGA	LOS (days)		*P*
				Mean	sd	
Fat stores						<0·001
0	207	53·2	4·8	12·1	6·9	
1	146	37·5	6·7	12·4	7·1	
2	29	7·4	11·5	12·6	6·8	
3	7	1·9	14·1	14·1	7·0	
Muscle status						<0·001
0	67	17·2	4·7	12·2	7·1	
1	289	74·3	5·6	12·5	7·3	
2	28	7·3	10·9	12·7	7·2	
3	5	1·2	13·8	13·2	7·4	
Fluid status						
0	299	76·9	5·3	12·1	7·2	<0·001
1	60	15·4	6·4	13·3	7·1	
2	25	6·4	13·5	13·7	6·9	
3	5	1·3	15·3	16·4	7·2	

For patients who stayed fewer than 30 d in the hospital, 1·0 % (*n* 4) of patients had stayed in the ICU, and these patients had a PG-SGA score of 7·1; in contrast, 99·0 % (*n* 385) of patients had not stayed in the ICU, and these patients had a PG-SGA score of 5·5. Patients who stayed in the ICU had a longer LOS (18·9 (sd 7·9) d) than patients who did not stay in the ICU (11·6 (sd 7·1) d) (Table 6). In our study, LOS was not influenced by health insurance (Table 7). The relationship and correlation between LOS and clinical factors are displayed in Table 8. The results revealed a significant relationship between LOS with PG-SGA (exp (B): 1·437, 95 % CI 1·365, 2·528), NRS 2002 (exp (B): 1·274, 95 % CI 1·237, 2·354) and TNM classification (OR: exp (B): 1·396, 95 % CI 1·252, 2·785).

**Table 6.** Patient-Generated Subjective Global Assessment (PG-SGA) and length of stay (LOS) according to ICU stay (Numbers and percentages; mean values and standard deviations)

ICU Stay	*n*	%	PG-SGA	LOS (days)		*P*
				Mean	sd	
Yes	4	1·0	7·1	18·9	7·9	<0·001
No	385	99·0	5·5	11·6	7·1	

**Table 7.** Patient-Generated Subjective Global Assessment (PG-SGA) and length of stay (LOS) according to health insurance (Numbers and percentages; mean values and standard deviations)

Health insurance	*n*	%	PG-SGA	LOS (days)		*P*
				Mean	sd	
No	27	6·9	6·3	12·3	7·1	0·513
Yes	362	93·1	6·5	12·5	7·5	

**Table 8.** Relationship and correlation between length of stay and clinical factors by multivariate logistic regression analyses in lung cancer patients (Coefficients and 95 % confidence intervals)

	*r*	Exp (B)	95 % CI	*P*
PG-SGA	0·327	1·437	1·365, 2·528	<0·001
NRS 2002	0·216	1·274	1·237, 2·354	<0·001
BMI	0·009	1·006	0·687, 1·439	0·465
TNM classification	0·314	1·396	1·252, 2·785	<0·001
Health insurance	–0·013	0·987	0·579, 1·265	0·513

PG-SGA, Patient-Generated Subjective Global Assessment; NRS 2002, Nutritional Risk Screening 2002; TNM, tumour node metastasis.

## Discussion

As reported by latest cancer research, malnutrition is associated with a high incidence (50–80 %), as estimated by PG-SGA^(12,13)^. There are a greater number of variables in PG-SGA than NRS 2002 to assess nutritional condition, like variables detecting symptoms possibly interfering with patient physical activity or food intake evaluation. As a valuable nutrition assessment tool, the PG-SGA requires that physical examination of patients should be performed by registered dietitians, skilled professional doctors or nurses. In this study, we examined the validity of the current version of the PG-SGA in predicting LOS among Northern Chinese patients with lung cancer. This research meets the urgent need to examine the validity of this widely used nutritional tool in Northern China and might profoundly impact the comprehensive nutritional management and assessment of cancer population in Northern China.

In addition, our results suggested that over 80 % of patients experienced malnutrition estimated PG-SGA, while 36·2 % of the overall samples were associated with nutritional risk by NRS 2002. The present data of cancer patients showed that poorer PG-SGA and NRS 2002 scores at admission and recent weight loss within 1 and 6 months predicted a longer LOS, which is consistent with other studies^(14–16)^. Prolonged LOS was also observed in underweight cases based on BMI. Relative to NRS 2002 and PG-SGA scores as well as the latest weight loss in the past 1 month, BMI showed a low predictive value, specificity and sensitivity in the prediction of LOS. The weight value of BMI did not change obviously, which may be related to the oedema that the patients had^(17)^. Our results showed that over 10 % cases experienced ascites and/or oedema, which suggested that the predictive significance of BMI and body mass may be biased to the greater values. Our findings are consistent with Ravasco’s finding that when compared with standard methods, BMI does not have a high sensitivity, specificity or predictive value^(18)^.

LOS can be used to predict patient prognostic outcome and is commonly utilised as the outcome marker^(19,20)^. Some variables can serve to determine LOS, such as patient-associated parameters, like age, diagnosis or treatment in hospital (such as surgery, chemotherapy and radiotherapy). However, malnutrition is significantly related to the prolonged LOS among cancer cases^(21–24)^. Our study also found that 6·9 % of the patients did not have health insurance, but this factor did not affect their LOS. As the economy has improved, the medical treatment of Northern Chinese patients has not been obviously influenced by medical insurance. We also discovered that the results of the PG-SGA were related to ICU stay. Patients who stayed in the ICU had poorer nutritional status and a longer LOS. Furthermore, the prediction of LOS is suggested as a vital task for the medical team to efficiently manage hospital resources and establish the sufficient health care plan.

Compared with cases with no nutritional risk, the NRS 2002 estimated that the prolonged LOS was seen in cases with a nutritional risk. Our findings from NRS 2002 and PG-SGA showed close relationship with the discharge time. We found that nutritional status could predict the LOS and hospitalisation expenses. Undernutrition has been associated with TNM classification, results of physical examinations (like fluid status, muscle status, fat reserves), longer LOS, increased overall expenses and ICU stay. It is recommended that nutritional screening should be conducted upon admission for detecting the candidate cases requiring nutritional intervention. As a result, determining cancer case nutritional status, especially in Northern China, is important, so as to provide reasonable interventions as early as possible. After nutrition screening and nutrition supplementation, tumour patients in Northern China may be more tolerant to tumour treatment. Thus, the effective rate of antitumour therapy and quality of life can be improved.

LOS is affected by numerous factors among cases who stayed in hospital for more than 30 d. Only patients hospitalised for 30 d or less were analysed in our study, and this cut-off has been previously used by Mendes^(25)^. It must be recognised that exclusion criteria had certain influence on external result validity. Future data will be used for determining the association of patient prognostic outcome with nutritional status based on NRS 2002 and PG-SGA.

To sum up, our results verified that malnutrition or nutritional risk estimated using NRS 2002, PG-SGA, or latest weight loss was related to the prolonged LOS. These findings suggest that more efforts are needed for improving the nutritional conditions in LUAD inpatients in Northern China. Moreover, it is extremely important to define approaches for efficiently preventing and treating malnutrition in hospital setting, so as to shorten LOS, improve patient life quality and promote the therapeutic efficacy.
