# Patient-Generated Subjective Global Assessment (PG-SGA) predicts length of hospital stay in lung adenocarcinoma patients – CORRIGENDUM

**DOI:** 10.1017/S0007114521005249

**Published:** 2022-05-28

**Authors:** Jilu Lang, Yanan Shao, Jiehao Liao, Jia Chen, Xuewen Zhou, Rong Deng, Wei-Jan Wang, Xian Sun

This article was published with incorrect corresponding author details.

Currently reads: * Corresponding author: Xian Sun, email sunshine1d1@126.com

This should read: * Corresponding authors: Xian Sun, email sunshine1d1@126.com and Wei-Jan Wang, email: cvcsky@gmail.com

