# Patient-Generated Subjective Global Assessment (PG-SGA) predicts length of hospital stay in lung adenocarcinoma patients – CORRIGENDUM

**DOI:** 10.1017/S0007114521004268

**Published:** 2022-05-28

**Authors:** Jilu Lang, Yanan Shao, Jiehao Liao, Jia Chen, Xuewen Zhou, Rong Deng, Wei-Jan Wang, Xian Sun

The authors apologise for a misspelling of the seventh author’s name: Wei-Jian Wang should instead read Wei-Jan Wang.

Also, the following financial support statement was erroneously omitted from the acknowledgements section:

This research was supported by China Medical University (CMU109-MF-55 to W.-J.W).

